# Effect of a first responder on survival outcomes after out-of-hospital cardiac arrest occurs during a period of exercise in a public place

**DOI:** 10.1371/journal.pone.0193361

**Published:** 2018-02-28

**Authors:** Seo Young Ko, Young Sun Ro, Sang Do Shin, Kyoung Jun Song, Ki Jeong Hong, So Yeon Kong

**Affiliations:** 1 Department of Emergency Medicine, Seoul National University College of Medicine, Seoul, Korea; 2 Laboratory of Emergency Medical Services, Seoul National University Hospital Biomedical Research Institute, Seoul, Korea; 3 Department of Emergency Medicine, Seoul National University Boramae Medical Center, Seoul, Korea; Osaka University Graduate School of Medicine, JAPAN

## Abstract

**Introduction:**

The deployment of first responders in a public place is one of the interventions that is used for increasing bystander cardiopulmonary resuscitation (CPR) of out-of-hospital cardiac arrests (OHCA). We studied the association between the presence of a first responder and the survival of OHCA that occurred during a period of exercise in a public place.

**Methods:**

All of the adult OHCAs of a presumed cardiac etiology that occurred during a period of exercise in a public place and that were witnessed by a bystander between 2013 and 2015 were analyzed. The main exposure of interest was the characteristics of the bystander (first responder vs. layperson). The endpoints were the provision of bystander CPR and good neurological recovery. Multivariable logistic regression analysis, adjusting for patient-environment and prehospital factors, was performed.

**Results:**

A total of 870 patients had a cardiac arrest during a period of exercise in a public place, and 58 (6.7%) patients were witnessed by the first responder. The OHCAs witnessed by first responders were more likely to result in bystander CPR than those witnessed by laypersons (89.7% vs. 75.4%, *p* = 0.01, adjusted OR (95% CI): 3.51 (1.44–8.55)). In terms of good neurological recovery, the OHCAs witnessed by first responders had a higher likelihood than the patients witnessed by laypersons (37.9% vs, 24.0%, *p* = 0.02, adjusted OR (95% CI): 2.92 (1.33–6.40)).

**Conclusion:**

The OHCAs occurred during a period of exercise in a public place and whom first responders witnessed were more likely to receive bystander CPR and to have a neurologically intact survival.

## Introduction

Out-of-hospital cardiac arrest (OHCA), which has a high incidence and low survival rate, is one of the most important public health issues [1]. In particular, OHCA that occurs during a period of exercise has been frequently highlighted by the media when the victim is a famous sports athlete and collapsed during a game. OHCA that occurs during a period of exercise is not as frequent as cardiac arrest that occurs during other activities, in which the proportions vary from 2% to 12% according to the communities [2–4]. However, the majority of OHCAs that occur during a period of exercise occurred in a relatively younger and physically active population [2–5]. Therefore, the burden of disease of OHCA that occurs during a period of exercise, including economic costs, are enormous, although the prognosis and survival outcomes are relatively better in these patients than in those who suffer from an arrest while engaging in other activities [2–6].

The majority of OHCAs that occur during a period of exercise were witnessed and had shockable rhythm for primary cardiac rhythm at the scene. Therefore, providing early bystander cardiopulmonary resuscitation (CPR) and defibrillation is crucial for the enhancement of neurological and survival outcomes [2–4, 6]. Several evidence-based strategies were developed and implemented to increase bystander CPR and to shorten the time from collapse to first chest compression and defibrillation, such as a mass CPR training program, dispatcher-assisted bystander CPR, mobile-phone dispatch of a trained layperson, and public access defibrillation (PAD) in a public place or in sports facilities [7–9].

One of these strategies is the designation and training of first responders; by designating and deploying well-trained first responders who can easily identify cardiac arrest that occurs during exercise, they would be able to quickly provide CPR and defibrillation when a cardiac arrest occurs and increase patient survival and favorable neurologic outcomes [7, 10–12]. However, the effect of the designation of and training program for first responders on the survival outcomes for OHCA that occurs during a period exercise in a public place has not yet been elucidated.

We hypothesize that a cardiac arrest patient whose arrest is witnessed by the first responder would be associated with a higher likelihood of the provision of bystander CPR and neurologically intact survival after OHCA than a patient whose arrest is witnessed by laypersons. The aim of this study was to evaluate the association between the characteristics of the witnessing bystanders (first responder vs. laypersons) and the provision of bystander CPR for OHCA patients who undergo an incident during a period of exercise in a public place.

## Methods

### Study design, setting, and data source

In Korea, the regulations of the national Emergency Medical Services (EMS) Act, which was implemented in 2004, designated first responders to encourage first aid for the citizens’ health and safety. First responders include firefighters, police officers, sports facility managers and sports instructors, safety guards of national parks, lifeguards, school health teachers, workplace safety employees, managers of nursing homes, public transportation vehicle drivers, and apartment safety guards in towns with more than 500 houses. Since 2005, first responders have been required to complete regular CPR and AED training every year via at least one two-hour course. The Ministry of Health and Welfare financially support the CPR and AED training program according to the EMS Act, and 112,935 first responders participated in the CPR training course from 2013 to 2015 (of the approximately 50 million persons in the population). Most places where first responders work or live are also mandatory sites for PAD programs, as designated by the EMS Act [12].

The EMS system is exclusively operated by the National Fire Department. EMS providers can perform CPR with an automated external defibrillator (AED) and cannot stop CPR unless the patient returns to having spontaneous circulation in the field or during transport to a hospital. All EMS-assessed patients are transported to an emergency department (ED). EMS providers should perform CPR for all EMS-assessed OHCA patients unless there are definitive signs of death that are confirmed by direct medical control with a physician. There are approximately 460 EDs, which are designated by the Ministry of Health and Welfare, and they generally perform acute cardiac care and post-resuscitation care in accordance with the International Standard Guidelines [13].

This is a cross-sectional study that uses a nationwide, prospective registry of OHCAs in Korea. The data were retrieved from the following four sources: EMS run sheets for basic ambulance operation information, EMS cardiac arrest registry and dispatcher CPR registry for the Utstein factors, and the National OHCA registry for hospital care and outcomes, which is extracted from hospital medical records by the Korea Centers for Disease Control and Prevention (CDC). The EMS providers record the EMS run sheets and cardiac arrest registry for all of the EMS-assessed OHCAs. The medical record reviewers from the Korea CDC (approximately 15 reviewers) extracted clinical information using structured forms based on the Utstein template from the medical records of all OHCA patients who were transported to EDs by EMS providers [14]. A quality management committee composed of representatives from the fire department, emergency physicians, epidemiologists, statistical experts, and medical record review experts ensured the quality of the medical record review processes. The quality management committee educated all of the medical record reviewers prior to joining the project, provided a standard manual for data abstraction, provided feedback to the reviewers on a monthly basis, and provided consultations in equivocal cases, as needed. A detailed description of Korea EMS system and quality control of the National OHCA registry are described in earlier studies [12, 13, 15, 16].

### Study population

This study included all EMS-treated patients with OHCAs of a presumed cardiac etiology who were 18 years of age or older on the day of the incident and whose event occurred during a period exercise in a public place and was witnessed by laypersons or first responders between January 2013 and December 2015. Information on the place of arrest was identified by EMS providers at the scene, and information on the specific activity in which the patient engaged at the time of, or immediately prior to, the arrest was ascertained from the medical record review. The OHCA during a period exercise in a public place was defined as the physical activity at the time of the incident and was one of the following: bicycling, conditioning exercises, dancing, fishing and hunting, engaging in sports, walking, running, water activities, and winter activities [2, 17]. Cases that had missing information regarding the neurological status at hospital discharge were excluded.

### Main outcomes

The primary outcome of this study was the provision of bystander CPR, as identified by EMS providers at the scene. The secondary outcomes were a good neurological recovery and survival at discharge from the hospital, as identified by a medical record review. Good neurological recovery was recorded if the patient had a cerebral performance category score of 1 (good cerebral performance) or 2 (moderate cerebral disability; able to perform daily activities independently).

### Measurements

The main exposure of interest was the bystanders, which was separated into two categories: (1) the first responders included police officers, sports facility managers and sports instructors, and other first responders who were designated by the EMS Act, and (2) laypersons included family members, friends or colleagues, and other laypersons, including nearby strangers who were not first responders. EMS providers classified and recorded the characteristics of bystanders (laypersons or first responders), and if the bystanders were laypersons, then they collected the relationship between the bystanders and the victim at the scene (family members, friends, or strangers).

We collected information on the age, gender, past medical history (hypertension, diabetes mellitus, heart disease, and stroke), residential area (metropolitan city), the metabolic equivalent of task (MET) score of the physical activities, time and season of the arrest, bystander CPR, time intervals from arrest to initial chest compression, type of primary cardiac rhythm identified at the scene (shockable or nonshockable rhythms), pre-hospital defibrillation by bystanders or EMS providers, response time interval from the call to the arrival of the ambulance at the scene, ROSC upon arrival in the ED, and post-resuscitation care.

### Statistical analysis

To determine the associations of the characteristics of bystander (first responders vs. laypersons) with the study outcomes, adjusted odds ratios (ORs) with 95% confidence intervals (95% CIs) of the study endpoints were calculated using multivariable logistic regression analysis. The model was adjusted for potential confounders, including patient-environment factors (age (adults vs. elderly), gender, past medical history (hypertension, diabetes mellitus, heart disease, and stroke), residential area (metropolitan city vs. urban and rural area), MET score of exercise at the time of the arrest (0–3 vs. 3–6 vs. 6–50), and time and season of the arrest) and EMS factors (type of primary cardiac rhythm on ECG identified at the scene (shockable vs. nonshockable rhythms) and EMS response time (<8 min vs. ≥8 min)). All of the variables in the final model were assessed for multicollinearity, which was not detected in this analysis.

All of the statistical analyses were performed using SAS software, version 9.4 (SAS institute Inc., Cary, NC, USA).

### Ethics statements

This study complied with the Declaration of Helsinki, and its protocol was approved by the Seoul National University Hospital Institutional Review Board with a waiver of informed consent (IRB No.: 1103-153-357).

## Results

### Demographic findings

**Fig 1:**
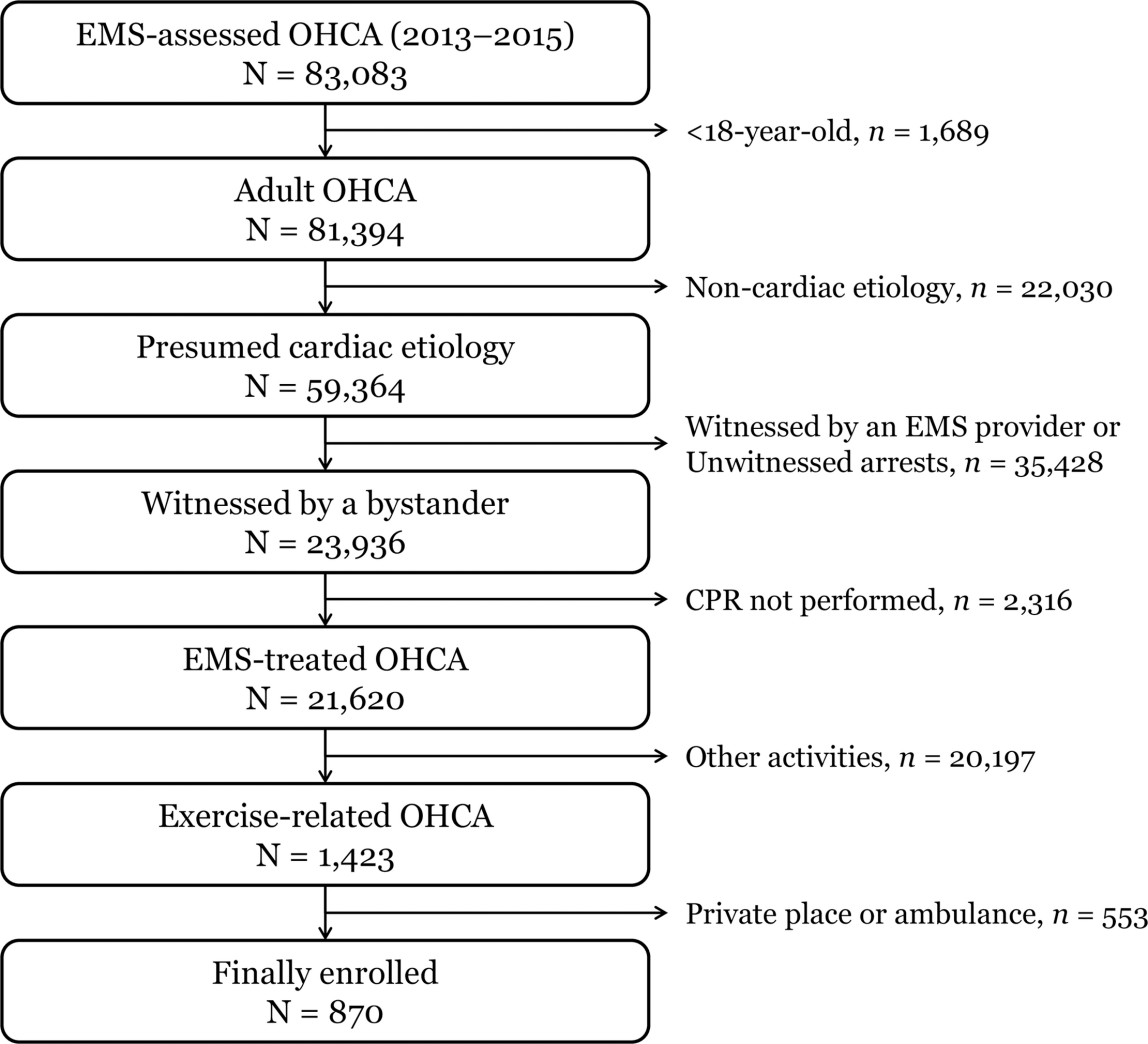
Study population. EMS: emergency medical service; OHCA: out-of-hospital cardiac arrest; CPR: cardiopulmonary resuscitation.

Among the 83,083 EMS-assessed patients who underwent OHCAs, 870 patients were included in the final analysis. We excluded patients who were younger than 18 years of age (n = 1,689), who had a non-cardiac etiology (n = 22,030), whose arrest was not witnessed by bystanders (n = 35,428), who did not receive resuscitation efforts by EMS providers (n = 2,316), whose arrest occurred during other activities than exercise (n = 20,197), and whose arrest occurred in a private place or ambulance (n = 553; Fig 1).

Of the 870 eligible study patients, 58 (6.7%) patients were witnessed by first responders and 812 (93.3%) were witnessed by laypersons. The provision of bystander CPR and defibrillation were more frequent in the first responder group than the layperson group (CPR: 89.7% vs. 75.4%, *p* = 0.01; defibrillation: 10.3% vs. 3.3%, *p* = 0.02). Shockable cardiac rhythm presented at the scene was shown in 50.0% of the first responder group and 55.9% of the layperson group (*p* = 0.38), and survival to discharge rates were 37.9% and 24.0% in the first responder and the layperson groups (*p* = 0.08), respectively. A good neurological recovery rate was higher in the first responder group than in the layperson group (41.4% vs. 30.4%, *p* = 0.02) (Table 1).

**Table 1:** Demographic and clinical characteristics of the bystanders.

		Total		First responder		Layperson		*p*-value
		N	%	N	%	N	%	
**Total**		870		58	6.7	812		
**Gender**								0.36
	**Male**	754	86.7	48	82.8	706	86.9	
**Age (years)**								0.95
	**18–64**	582	66.9	39	67.2	543	66.9	
	**≥65**	288	33.1	19	32.8	269	33.1	
	**Median (IQR)**	57.9 (49.8–68.8)		55.2 (43.7–68.0)		57.9 (50.3–69.2)		<0.01
**Past medical history**								
	**Diabetes mellitus**	138	15.9	7	12.1	131	16.1	0.41
	**Hypertension**	272	31.3	17	29.3	255	31.4	0.74
	**Heart disease**	176	20.2	10	17.2	166	20.4	0.56
	**Stroke**	38	4.4	1	1.7	37	4.6	0.51
**Residential area**								0.22
	**Metropolitan**	413	47.5	32	55.2	381	46.9	
**MET score**								<0.01
	**0–3**	104	12.0	5	8.6	99	12.2	
	**3–6**	426	49.0	43	74.1	383	47.2	
	**≥6**	340	39.1	10	17.2	330	40.6	
	**Median (IQR)**	4.8 (3.5–7.0)		3.5 (3.5–5.5)		5.0 (3.5–7.0)		<0.01
**Time of arrest**								0.57
	**Daytime**	671	77.1	43	74.1	628	77.3	
**Season of arrest**								0.83
	**Spring**	246	28.3	19	32.8	227	28.0	
	**Summer**	191	22.0	13	22.4	178	21.9	
	**Autumn**	235	27.0	15	25.9	220	27.1	
	**Winter**	198	22.8	11	19.0	187	23.0	
**Bystander CPR**								0.01
	**Chest compression**	664	76.3	52	89.7	612	75.4	
**Time from arrest to initial chest compression**								
	**Available value, n**	746	85.7	51	87.9	695	85.6	
	**Median (IQR), min**	2 (0–7)		0 (0–3)		2 (0–7)		<0.01
**Primary cardiac rhythm at the scene**								
	**Shockable**	483	55.5	29	50.0	454	55.9	0.38
**Prehospital defibrillation**								
	**By an EMS provider**	541	62.2	34	58.6	507	62.4	0.56
	**By a bystander**	33	3.8	6	10.3	27	3.3	0.02
**EMS response time (min)**								<0.01
	**0–4**	63	7.2	6	10.3	57	7.0	
	**4–8**	339	39.0	32	55.2	307	37.8	
	**8–12**	210	24.1	13	22.4	197	24.3	
	**≥12**	258	29.7	7	12.1	251	30.9	
	**Median (IQR)**	8 (5–13)		7 (5–8)		8 (6–14)		0.82
**Post-resuscitation care**								
	**Reperfusion therapy**	133	15.3	13	22.4	120	14.8	0.12
	**Therapeutic hypothermia**	72	8.3	8	13.8	64	7.9	0.13
**Survival outcomes**								
	**ROSC on arrival in the ED**	262	30.1	26	44.8	236	29.1	0.01
	**Survival to discharge**	271	31.1	24	41.4	247	30.4	0.08
	**Good neurological recovery**	217	24.9	22	37.9	195	24.0	0.02

MET: metabolic equivalent of task; IQR: interquartile range; CPR: cardiopulmonary resuscitation; EMS: emergency medical services; ED: emergency department; ROSC: return of spontaneous circulation.

Among the 58 OHCAs that occurred during a period exercise in a public place and whose arrest was witnessed by the first responder, half (n = 29) of them were witnessed by sports facility managers or sports instructors, while a quarter (n = 14) of them were witnessed by police officers. Good neurological recovery rates were 48.3% in the sports facility manager group, 21.4% in the police officer group, and 33.3% in the other first responder group. Among the 812 OHCAs that were witnessed by laypersons, 17.2% were witnessed by family members, and 34.7% were witnessed by friends or colleagues. Bystander CPR was performed in 58.6% of the OHCAs witnessed by family members, 83.7% of those by friends, and 75.4% of those by other laypersons. A good neurological recovery rate was evidenced in 15.7% in the family members group, 30.9% in the friends group, and 22.1% in the other laypersons group (Table 2).

**Table 2:** Study outcomes according to the bystanders.

			Total	Bystander CPR		Bystander defibrillation		ROSC on arrival in the ED		Survival to discharge		Good neurological recovery	
			N	N	%	N	%	N	%	N	%	N	%
**Total**			870	664	76.3	33	3.8	262	30.1	271	31.1	217	24.9
**First responder**													
	**Total**		58	52	89.7	6	10.3	26	44.8	24	41.4	22	37.9
		**Sport facility managers**	29	26	89.7	3	10.3	15	51.7	15	51.7	14	48.3
		**Police officers**	14	11	78.6	1	7.1	5	35.7	4	28.6	3	21.4
		**Other first responders**	15	15	100.0	2	13.3	6	40.0	5	33.3	5	33.3
**Layperson**													
	**Total**		812	612	75.4	27	3.3	236	29.1	247	30.4	195	24.0
		**Family members**	140	82	58.6	2	1.4	24	17.1	29	20.7	22	15.7
		**Friends or colleagues**	282	236	83.7	9	3.2	109	38.7	106	37.6	87	30.9
		**Other laypersons**	390	294	75.4	16	4.1	103	26.4	112	28.7	86	22.1

CPR: cardiopulmonary resuscitation; ED: emergency department; ROSC: return of spontaneous circulation.

### First responder vs. layperson in survival outcomes

Multivariable analysis showed that the OHCA patients whose event was witnessed by a first responder were more likely to receive bystander CPR and have good neurological recovery compared to patients whose arrest was witnessed by a layperson. Adjusting for the patient-environment and EMS factors, the adjusted ORs (95% CIs) for the provision of bystander CPR and good neurological recovery in the first responder group compared with the laypersons group were 3.51 (1.44–8.55, *p* <0.01) and 2.92 (1.33–6.40, *p* <0.01), respectively. In terms of survival to hospital discharge, there was no significant difference between the two groups (adjusted ORs (95% CI): 2.00 (0.98–4.07); *p* = 0.06) (Table 3). The full results of the multivariable analysis are shown in the S1 Table.

**Table 3:** Associations between bystanders and study outcomes for study population.

		Total	Outcome		Unadjusted	Adjusted*
		N	N	%	OR (95% CI)	OR (95% CI)
**Provision of bystander CPR**						
	**Laypersons**	812	612	75.4	1.00	1.00
	**First responders**	58	52	89.7	2.83 (1.20–6.69)	3.51 (1.44–8.55)
**Survival to discharge**						
	**Laypersons**	812	247	30.4	1.00	1.00
	**First responders**	58	24	41.4	1.62 (0.94–2.78)	2.00 (0.98–4.07)
**Good neurological recovery**						
	**Laypersons**	812	195	24.0	1.00	1.00
	**First responders**	58	22	37.9	1.93 (1.11–3.37)	2.92 (1.33–6.40)

OR: odds ratio; 95% CI: 95% confidence interval; CPR: cardiopulmonary resuscitation

*Adjusted for patient–environment factors (age, gender, past medical history (hypertension, diabetes mellitus, heart disease, and stroke), residential area, MET score of exercise at the time of the arrest, and time and season of the arrest) and EMS factors (EMS response time and primary cardiac rhythm at the scene).

## Discussion

We observed significant associations between the bystander characteristics (first responder or layperson) with a subsequent prognosis for the OHCA patients whose event was witnessed and occurred during a period of exercise in a public place. Deployment of a trained first responder increased the frequency of the provision of bystander CPR and neurologically intact survival. Our results emphasize that the designation and deployment of a well-trained first responder in public places would be a strong intervention for the neurological recovery of patients whose cardiac arrest occurs during a period exercise.

### Designation and deployment of the first responder

Prognosis and survival outcomes after cardiac arrest are affected by the time from the collapse to the first chest compression. Therefore, many evidence-based strategies were developed and implemented for providing timely high-quality bystander CPR. While the bystander CPR rate has improved considerably in the last several years, the provision of bystander defibrillation remained low and whether the quality of bystander CPR has improved is also doubtful [18–20]. Designating well-trained first responders by the national government and deploying them where cardiac arrest frequently occurs should aim to provide high-quality CPR and defibrillation in a timely manner to patients who suffer from cardiac arrest [21, 22]. Among the OHCAs that occurred during a period of exercise in a public place in this study, the patients who were witnessed by a first responder had a higher likelihood of receiving bystander CPR (89.7% vs. 75.4%) and defibrillation (10.3% vs. 3.3%) and were more likely to receive early initial chest compression (median 0 min vs. 2 min) compared to those who were witnessed by a layperson. This study demonstrated an approximately threefold benefit for a favorable neurological recovery (adjusted OR: 2.92 (1.33–6.40)) for OHCAs witnessed by a first responder compared with those witnessed by a layperson. In a previous study, the best outcomes in bystander CPR and defibrillation have been observed in settings where a network of well-trained first responders and EMS providers responded rapidly and appropriately to an OHCA patient [21, 22]. However, the survival outcomes of the OHCAs witnessed by the first responders varied widely depending on the types of responders, which may be due to the various skill sets of different first responders. By increasing the number of first responders designated by law to be continuously trained and educated to maintain knowledge and skills, deploying them in places with a high incidence of cardiac arrest, and building a network of EMS providers, the number of patients who receive timely high-quality CPR and defibrillators will increase, and this will eventually lead to better survival and neurological outcomes.

### Cardiac arrests occurred during period exercise in public place

Since a healthy lifestyle that includes daily physical activity, weight management, and a balanced diet is recommended to reduce the risk of cardiovascular disease, the population that is enjoying physical exercise, including jogging and physical training in sports facilities, is increasing [23]. However, it is well known that the risk of an acute cardiac event, including sudden cardiac arrest, is transiently increased during or shortly after vigorous physical activity [24, 25]. OHCAs witnessed by laypersons in this study had larger METs of physical activities and longer EMS response time. In Korea, vigorous physical activity such as mountain climbing is common, and OHCA occurring during climbing is more likely to be witnessed by a layperson than a first responder and to have longer EMS response time. The type of bystander may be different depending on the place and type of physical activity of OHCA patients.

The most common cause of exercise-related arrest was acute coronary syndrome, and the majority of them had a shockable rhythm at the scene [4, 24, 26]. In this study, more than half of the study population had a shockable rhythm as primary cardiac rhythm at the scene and 66.0% had received defibrillation during prehospital CPR by an EMS provider or bystander. The study population must have been given a strong chance to achieve survival by others providing early CPR and defibrillation on them. The evidence-based strategies to shorten the time from the collapse to the first chest compression and defibrillation, including deployment of well-trained first responders, may have an impact on increasing the survival outcomes in this population.

### Limitations

This study has several limitations. First, the study population consisted of OHCA patients who underwent cardiac arrest that occurred during a period exercise, and we measured the physical activity of the patients at the time of, or immediately prior to, the cardiac arrest from the medical records. The information was limited compared to more objective measures of physical fitness, which could create a selection bias that can underestimate or overestimate the effect. Second, our nationwide OHCA database did not collect information regarding the number of bystanders who witnessed the cardiac arrest and whether the bystanders had the capacity to perform CPR, which would influence both the bystander characteristics and the study outcomes. Also the registry did not collect information on quality of bystander CPR including chest compression rate and depth and time to defibrillation. Third, this study was done in an EMS system with intermediate service level. Generalization of the study findings should be made with caution. Lastly, this study was an observational study using the nationwide OHCA registry and not a randomized controlled trial. Thus, there potentially may be significant biases that were not controlled. The registry captured all EMS-assessed OHCA, however, the OHCA patients who transported to the hospital by other methods was not enrolled in the registry.

## Conclusions

Out-of-hospital cardiac arrest patients who undergo cardiac arrest during a period of exercise in a public place and who were witnessed by first responders were more likely to receive bystander CPR and have a neurologically intact survival compared to patients who were witnessed by laypersons. Our results emphasized that the designation and deployment of first responder in public places would be an advantageous strategy for patients who have an OHCA that occurs during a period exercise in a public place.

## Supporting information

S1 Table Multivariable logistic regression model for study population. (DOCX)
