# Supplementary material for: Effect of a first responder on survival outcomes after out-of-hospital cardiac arrest occurs during a period of exercise in a public place
Source: PLoS One. 2018 Feb 28;13(2):e0193361. doi: 10.1371/journal.pone.0193361 (PMC5831003; doi:10.1371/journal.pone.0193361)
Supplement: S1 Table — (DOCX) [file pone.0193361.s001.docx]

| Supplementary table. Multivariable logistic regression model for study population | | | | | | | |
| --- | --- | --- | --- | --- | --- | --- | --- |
|  | | Provision of bystander CPR | | Survival to discharge | | Good neurological recovery | |
|  |  | Model 1 | Model 2 | Model 1 | Model 2 | Model 1 | Model 2 |
|  |  | OR (95% CI) | OR (95% CI) | OR (95% CI) | OR (95% CI) | OR (95% CI) | OR (95% CI) |
| Type of bystander | |  |  |  |  |  |  |
|  | Laypersons | 1.00 | 1.00 | 1.00 | 1.00 | 1.00 | 1.00 |
|  | First responders | 3.07 (1.27–7.41) | 3.51 (1.44–8.55) | 1.53 (0.86–2.74) | 2.00 (0.98–4.07) | 1.90 (1.04–3.44) | 2.92 (1.33–6.40) |
| MET score | |  |  |  |  |  |  |
|  | 0–3 (vs. 3–6) | 0.58 (0.36–0.94) | 0.60 (0.37–0.98) | 0.49 (0.28–0.86) | 0.59 (0.31–1.12) | 0.43 (0.23–0.82) | 0.49 (0.23–1.02) |
|  | ≥6 (vs. 3–6) | 1.40 (0.94–2.07) | 1.34 (0.89–2.02) | 0.69 (0.49–0.97) | 1.04 (0.69–1.55) | 0.77 (0.54–1.11) | 1.25 (0.81–1.93) |
| Gender | |  |  |  |  |  |  |
|  | Female (vs. male) | 0.86 (0.54–1.38) | 1.00 (0.61–1.62) | 0.57 (0.34–0.96) | 1.07 (0.58–1.97) | 0.45 (0.24–0.83) | 0.86 (0.42–1.75) |
| Age | |  |  |  |  |  |  |
|  | 18–64 (vs. ≥65) | 2.30 (1.59–3.33) | 2.03 (1.38–2.96) | 2.94 (1.99–4.35) | 2.24 (1.42–3.53) | 3.08 (1.99–4.77) | 2.35 (1.42–3.91) |
| Residential area | |  |  |  |  |  |  |
|  | Urban, rural (vs. metropolitan city) | 0.91 (0.65–1.26) | 0.87 (0.62–1.22) | 0.66 (0.49–0.90) | 0.83 (0.58–1.18) | 0.70 (0.50–0.97) | 0.91 (0.61–1.34) |
| Time of arrest | |  |  |  |  |  |  |
|  | Nighttime (vs. daytime) | 0.76 (0.51–1.14) | 0.79 (0.52–1.18) | 1.46 (1.03–2.09) | 1.26 (0.83–1.91) | 1.51 (1.04–2.20) | 1.24 (0.79–1.92) |
| Season of arrest | |  |  |  |  |  |  |
|  | Spring (vs. winter) | 1.06 (0.67–1.68) | 1.06 (0.67–1.69) | 0.70 (0.46–1.08) | 0.62 (0.38–1.02) | 0.91 (0.57–1.44) | 0.90 (0.52–1.55) |
|  | Summer (vs. winter) | 0.87 (0.54–1.41) | 0.82 (0.51–1.34) | 0.66 (0.42–1.03) | 0.68 (0.41–1.14) | 0.74 (0.45–1.21) | 0.87 (0.49–1.52) |
|  | Autumn (vs. winter) | 1.27 (0.79–2.05) | 1.19 (0.73–1.92) | 0.83 (0.54–1.27) | 0.73 (0.44–1.22) | 1.14 (0.72–1.80) | 1.14 (0.66–1.97) |
| Past medical history | |  |  |  |  |  |  |
|  | Diabetes mellitus | 1.41 (0.91–2.18) | 1.34 (0.86–2.09) | 1.25 (0.81–1.95) | 1.08 (0.65–1.79) | 1.58 (0.97–2.58) | 1.45 (0.83–2.54) |
|  | Hypertension | 1.02 (0.71–1.48) | 1.09 (0.75–1.59) | 0.72 (0.50–1.02) | 0.93 (0.62–1.40) | 0.72 (0.49–1.04) | 0.96 (0.62–1.48) |
|  | Heart disease | 0.77 (0.51–1.18) | 0.87 (0.57–1.33) | 0.63 (0.43–0.91) | 0.75 (0.48–1.16) | 0.53 (0.36–0.79) | 0.60 (0.37–0.96) |
|  | Stroke | 1.26 (0.61–2.58) | 1.46 (0.71–3.01) | 0.82 (0.38–1.78) | 1.03 (0.44–2.43) | 1.34 (0.52–3.46) | 1.73 (0.63–4.78) |
| EMS response time, min | |  |  |  |  |  |  |
|  | ≥8 min (vs. <8 min) |  | 1.53 (1.06–2.19) |  | 0.30 (0.21–0.44) |  | 0.26 (0.17–0.39) |
| Primary cardiac rhythm at the scene | | |  |  |  |  |  |
|  | Nonshockable (vs. shockable) |  | 0.49 (0.34–0.70) |  | 0.07 (0.04–0.12) |  | 0.04 (0.02–0.07) |
| OR: odds ratio; 95% CI: 95% confidence interval; CPR: cardiopulmonary resuscitation; MET: metabolic equivalent of task; EMS: emergency medical services | | | | | | | |
| *Model 1 adjusted for patient–environment factors (age, gender, past medical history (hypertension, diabetes mellitus, heart disease, and stroke), residential area, MET score of exercise at the time of the arrest, and time and season of the arrest). | | | | | | | |
| *Model 2 adjusted for patient–environment factors and EMS factors (EMS response time and primary cardiac rhythm at the scene). | | | | | | | |
